# Testing Pathological Variation of White Matter Tract in Adult Rats after Severe Spinal Cord Injury with MRI

**DOI:** 10.1155/2018/4068156

**Published:** 2018-11-11

**Authors:** Wei Song, Guiyun Song, Can Zhao, Xiaoguang Li, Xiaojiao Pei, Wen Zhao, Yudan Gao, Jia-Sheng Rao, Hongmei Duan, Zhaoyang Yang

**Affiliations:** ^1^Capital Medical University School of Rehabilitation Medicine, Beijing 100068, China; ^2^Institute of Rehabilitation Engineering, China Rehabilitation Research Centre, Beijing 100068, China; ^3^Department of Rehabilitation Evaluation, Beijing Bo'ai Hospital, China Rehabilitation Research Centre, Beijing 100068, China; ^4^Beijing Advanced Innovation Center for Biomedical Engineering, Beihang University, Beijing 100083, China; ^5^Beijing International Cooperation Bases for Science and Technology on Biomaterials and Neural Regeneration, Beijing Advanced Innovation Center for Big Data-Based Precision Medicine, Beihang University, Beijing 100083, China; ^6^Department of Measurement Control and Information Technology, School of Instrument Science and Opto-Electronic Engineering, Beihang University, Beijing 100083, China; ^7^Department of Neurobiology, School of Basic Medical Sciences, Capital Medical University, Beijing 100069, China; ^8^Department of Radiology, Beijing Chao-Yang Hospital, Capital Medical University, Beijing 100043, China; ^9^Beijing Key Laboratory for Biomaterials and Neural Regeneration, School of Biological Science and Medical Engineering, Beihang University, Beijing 100083, China

## Abstract

The purpose of this study was to assess the pathological variation in white matter tracts in the adult severe thoracic contusion spinal cord injury (SCI) rat models combined with in vivo magnetic resonance imaging (MRI), as well as the effect of spared white matter (WM) quantity on hindlimb motor function recovery. 7.0T MRI was conducted for all experimental animals before SCI and 1, 3, 7, and 14 days after SCI. The variation in the white matter tract in different regions of the spinal cord after SCI was examined by luxol fast blue (LFB) staining, NF200 immunochemistry, and diffusion tensor imaging (DTI) parameters, including fraction anisotropy, mean diffusivity, axial diffusion, and radial diffusivity. Meanwhile, Basso-Beattie-Bresnahan (BBB) open-field scoring was performed to evaluate the behavior of the paraplegic hind limbs. The quantitative analysis showed that spared white matter measures assessed by LFB and MRI had a close correlation (R^2^ = 0.8508). The percentage of spared white matter area was closely correlated with BBB score (R^2^ = 0.8460). After SCI, spared white matter in the spinal cord, especially the ventral column WM, played a critical role in motor function restoration. The results suggest that the first three days provides a key time window for SCI protection and treatment; spared white matter, especially in the ventral column, plays a key role in motor function recovery in rats. Additionally, DTI may be an important noninvasive technique to diagnose acute SCI degree as well as a tool to evaluate functional prognosis. During the transition from nerve protection toward clinical treatment after SCI, in vivo DTI may serve as an emerging noninvasive technique to diagnose acute SCI degree and predict the degree of spontaneous functional recovery after SCI.

## 1. Introduction

Spinal cord injury (SCI) may cause partial or total sensory and motor dysfunction beneath the injury interface, as well as the loss of autonomic nerve function [1, 2]. The pathological processes that appear after SCI are generally described in terms of the primary phase and secondary phase. Most primary phase lesions come from mechanical damage and directly result from the SCI and hemorrhage, and the gray matter is particularly vulnerable during this phase [3]. The secondary phase lesions that occur in rodents and mammals may last several hours or even several months and are accompanied by edema, hemorrhage, inflammation, and cytotoxic edema as the major symptoms [4–6], which may extend to the white matter area and lead to white matter degeneration and damage [7, 8]. Experimental SCI models are diverse and include contusion injury, compression injury, transection injury, and semitransection injury, among which contusion injury is considered most similar to the clinical condition [9]. As previously reported, the gray matter loss at the lesion site and surrounding areas does not last more than 24 h; however, the white matter loss may continue for over 1 week and spread from the lesion core to the distal end of the white matter tracts [2]. Previous research has demonstrated that spared white matter holds the key to motor functional recovery of hindlimbs after SCI, and it is closely correlated with functional restoration of the paralyzed hind limbs [10–12]. Experimental data have demonstrated that spared white matter of less than 5% resulted in a loss of weight-supporting ability in the rat hindlimbs; spared white matter of 12%-15% at the lesion center might maintain high-quality hind limb movements, such as consistent bilateral, hindlimb, weight-supported plantar steps, while consistent forelimb-hindlimb coordination and precise stepping (horizontal bar and beam walking) required contact between multiple fiber tracts [11, 13, 14]. Because white matter injury is the major cause of function loss after SCI [4, 12], many therapeutic strategies focus on the effects of secondary lesions to white matter.

In the clinic, to further reveal the correlation between the pathological processes of white matter impairment and behavioral function after SCI, a noninvasive in vivo assay technique is needed to assess the spatiotemporal variations in white matter after SCI. Diffusion tensor imaging (DTI) is an advanced MRI technique that is capable of indirectly assessing the integrity of in vivo microstructures by examining the diffusion of water molecules in biological tissues. Compared with conventional MRI, DTI has found wide application in testing tissues and structures of the central nervous system (CNS), such as the brain [15] and spinal cord [16–18]. At present, DTI is the only noninvasive technique used to observe neural fiber orientation in vivo. In myelinated axons of the CNS, free diffusion of water molecules is restricted by neuronal structure and thus displays anisotropy. DTI takes three vertical diffusion eigenvectors e1, e2, and e3 to describe such anisotropy and uses three corresponding eigenvalues *λ*1, *λ*2, and *λ*3 to reflect the diffusivity of water molecules, where axial diffusivity (AD) is parallel to the axonal direction and radial diffusivity (RD) is perpendicular to the axonal direction. As previously reported, fraction anisotropy (FA) and AD are closely correlated with axonal integrity [16, 19, 20], and RD reflects myelination integrity [16]. Meanwhile, the mean diffusivity (MD) could provide information about the state of edema in SCI and was sensitive to cysts in chronic SCI [21, 22]. Collectively, using DTI to test these parameters in the white matter enables the assessment of microstructural information in the white matter tissue [4, 16].

Research has shown that DTI, as a noninvasive in vivo imaging test, can not only test the white matter degree of damage [23–28] but also indirectly serve as a predictor of motor functional recovery after SCI [13, 18, 29]; however, few experiments have been conducted to spatiotemporally study the pathological process of white matter alterations after SCI using in vivo MRI, morphology and behavior in combination. In this study, using a severe contusion model and a combination of experimental techniques, we studied the spatiotemporal pathological process of white matter alterations after SCI, as well as the influence of spared white matter on motor function recovery. The study further demonstrated that the in vivo MRI technique can specifically evaluate and predict the spared white matter, in a quantitative manner, and spontaneous functional recovery after SCI, which may provide a theoretical foundation and offer technical evidence for adequate prediction and evaluation of SCI prognosis using noninvasive MRI technique in the clinic.

## 2. Materials and Methods

### 2.1. Animals Model Preparation

Thirty adult female Wistar rats, weighing 180-200 g and of SPF grade, were provided by the Experimental Animal Center of Capital Medical University. Equithesin was injected i.p. at 3 ml/kg to the rats for anesthesia. A sagittal incision was made at the lower dorsal part of the thoracic segment to expose the T7-T9 vertebral plate and spinous processes. A T8 vertebral plate was cut and removed under a surgical microscope to expose the intact dura. The rat was fixed on the base of an NYU impactor, with its T7 and T9 spinous processes held by a fixing device to fix the spinal cord. An impact stick of 2.5 mm in diameter and weighing 10 g was freely dropped from 50 mm high to contuse the rat spinal cord with 10 s intervals from dropping to lifting [11, 30]. The muscle layers and skin layers were sutured after contusion. Healthy rats were used for the normal control group. The experimental animals were carefully observed after the operation for their mental status, eating and drinking, and urination, as well as edema and ulcers. After the operation, penicillin was injected at 100000 units/time, successively for 7 days. The bladder was massaged 2-3 times each day until the bladder recovered its spontaneous urinary function. All of the above experiments were approved by the Animal Ethic Committee of Capital Medical University.

### 2.2. In Vivo MRI

Sodium amytal (1%) was injected i.p. at 5 ml/kg to anesthetize the rats. Before the operation and 1, 3, 7 and 14 days after the operation (n = 6, each group), a 7.0 T MRI scanner (Bruker, PharmaScan) with a transmit volume coil and a receive surface coil was used to obtain data. MRI scanning adopted the RARE sequence, with the following parameters: TR/TE = 3000/45 ms, matrix = 256 × 256, FOV = 37.6 mm × 37.6 mm, 20 slices, slice thickness = 1 mm with no gap, NEX = 6, resolution = 0.147 × 0.147 × 1 mm^3^, and scanning time = 9 min 44 s. DTI scanning adopted the SE-EPI sequence, with the following parameters: TR/TE = 5000/23 ms, matrix = 128 × 128, FOV = 18.8 mm × 18.8 mm, slice thickness = 1 mm, 30 gradient direction, 5 b0 images, b = 0 and 670 s/mm^2^, NEX = 4, and scanning time = 11 min 40 s. DTI data were processed and analyzed with MedINRIA software (http://www-sop.inria.fr/asclepios/software/MedINRIA/).

### 2.3. Quantification of Spared White Matter and DTI Data

The white matter of health animals were manually delineated in T2w images. The mean and SD of gray values from all white matter pixels was calculated based on the T2w image gradation histogram. Normalizing the intensity of all T2 images in injured groups is based on normal spinal cord images. Then a threshold was set at mean ± 2SD for the segmentation of the speared white matter in all normalized injured images. The segmented area was then used to calculate the spared white matter area percentage, i.e., spared white matter area percentage = number of pixels in the spared white matter /number of pixels in the whole cord.

**Figure 1:**
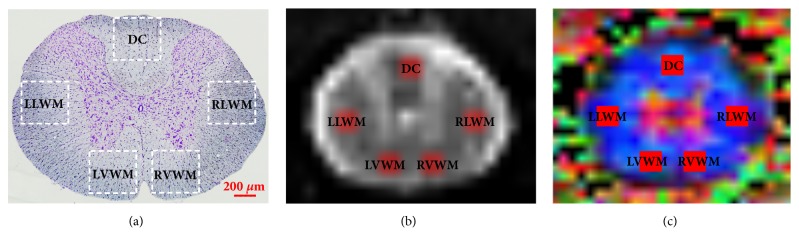
Set of different tractography ROI regions. (a) Pathological image. (b) MRI image. (c) Color FA map. DC (dorsal column), LLWM (left lateral white matter), RLWM (right lateral white matter), LVWM (left lateral white matter), and RVWM (right lateral white matter).

According to the anatomical images, the regions of interest (ROIs) at the lesion core were manually set at the dorsal column (DC), left lateral white matter (LLWM), right lateral white matter (RLWM), left ventral white matter (LVWM), and right ventral white matter (RVWM) (Figure 1), while trying to avoid the blank areas and the partial volume effect of cerebrospinal fluid. The DTI parameters, including FA, MD, AD, and RD values, in the ROIs were calculated and analyzed.

### 2.4. Behavioral Evaluation

Before the operation and 1, 3, 7, and 14 days after the operation, a double-blind evaluation of the Basso-Beattie-Bresnahan (BBB) open-field locomotion scale was performed for all experimental rats [11, 30]. At each time point, the average motor score of the bilateral hindlimbs was taken as the outcome value for each rat. Repeated measures analysis of variance (ANOVA) and Bonferroni post hoc analysis were adopted to determine the statistical significance of differences for multiple group comparisons [31, 32].

### 2.5. Sampling

Before the operation and at each time point after the operation, four or five experimental animals were arbitrarily chosen and sacrificed with an overdose anesthetic. Following cardiac perfusion with saline and 4% paraformaldehyde solution, a 2.0 cm long spinal cord segment, which included the lesion core, was excised and placed in 4% PFA for 6 h and then stored in 30% sucrose in 0.1 M PBS (pH 7.4) overnight. The spinal tissue, including the lesioned core, was embedded in O.C.T. compound (Sakura Tokyo, Japan), and coronal sections were continuously cut at 20 *μ*m with a Leica 1850 cryostat.

### 2.6. LFB Staining

LFB staining was used to test the myelin sheath of the white matter in the spinal cord [33, 34]. Tissue at 5 mm rostral to the lesion core (R5mm), the lesion core (LC), and 5 mm caudal to the lesion core (C5mm) were taken at each time point from the frozen, continuous coronal sections. The sections were first immersed in 60°C 1% LFB solution for 2 h and then washed with 95% alcohol and distilled water. After that, the sections were differentiated for 10 s with 0.05% lithium carbonate and subsequently with 70% alcohol until obvious distinction of gray matter and white matter appeared (which usually needed 30-60 s) and finally completely washed with distilled water. Next, the sections were immersed in 0.1% cresyl violet solution (prepared with 1% glacial acetic acid) for a 5 min reaction at 37°C. After washing with distilled water, the sections were washed successively with 70% alcohol and 95% alcohol until the Nissl bodies turned purple. The myelin sheath area percentage = LFB staining area / total section area × 100%.

Immunofluorescent staining: the immunofluorescent staining method has been published previously [31, 32]. The sections were incubated in 10% normal goat serum at 37°C for 30 min and then incubated in the primary antibody mouse anti-NF-200 (Abcam; 1:100) at 37°C for 30 min. The sections were then washed three times with 0.01 M phosphate-buffered saline (PBS, pH 7.4) and then incubated with goat anti-mouse fluorescence secondary antibody-Alex488 (green) (Invitrogen, 1:200) at room temperature for 30 min in the dark. The sections were coverslipped with Vectashield-mounting medium containing DAPI (Vector Laboratories) and examined under a fluorescence microscope (BX-51; Olympus, Tokyo, Japan).

Morphological analysis: morphological analysis and quantification were performed as described previously with modifications [32]. Axon counting method: an ROI region (294 *μ*m × 294 *μ*m, 86436 *μ*m^2^) was selected from each of the DC, LLWM, RLWM, LVWM, and RVWM regions of spinal cord sections. IPP6.0 software was used for NF-200+ counting under a high magnification using a counting frame [31, 32].

### 2.7. Statistical Analysis

Unless stated otherwise, data are presented as the mean ± SEM. The Shapiro–Wilk test was used for data normality analysis. Levene's test was used to test for homogeneity of variance. One-way ANOVA and Bonferroni analysis (multiple comparison for three groups) were conducted, and P < 0.05 was considered an indication of a statistically significant difference. Correlations between spared white matter percentage and BBB behavior was analyzed with the double-variant Pearson correlation analysis.

## 3. Results

### 3.1. The Quantity of Spared White Matter Myelin

**Figure 2:**
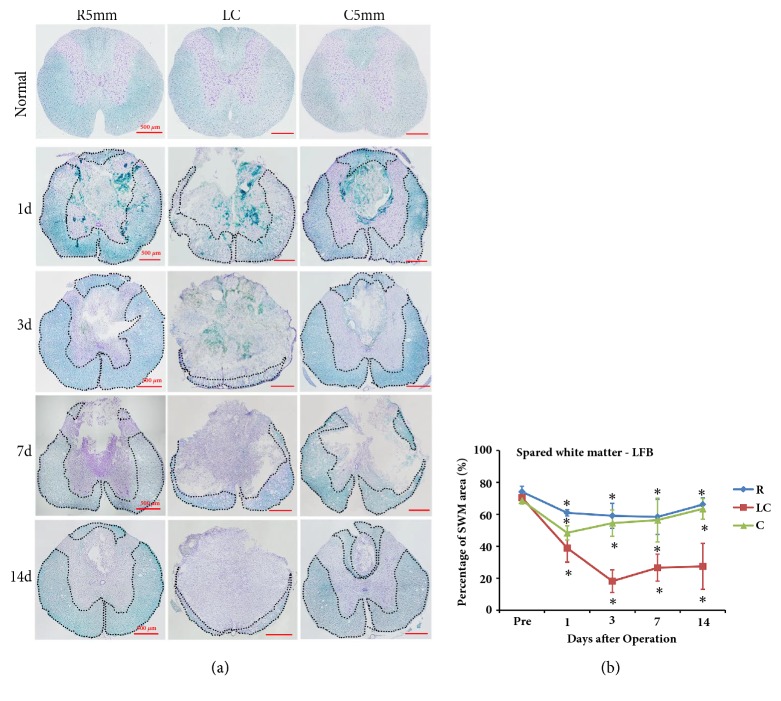
LFB and Nissl staining of coronal sections at the lesion core (LC), R5mm, and C5mm segments at each time point after SCI. The variation in the percentage of spared white matter area over time was observed. (a) LFB and Nissl staining of coronal sections at the lesion core, R5mm, and C5mm segments at each time point after SCI. The anatomic structure at the lesion core of the spinal cord was clearly changed, where white matter at the DC was greatly lost, gray matter was transformed severely, and a large cyst formed at the spinal cord center, which was filled with a large amount of denatured and dead neurons and disintegrated axons and myelin debris as well as inflammatory cells. The dotted line indicates spared white matter area. (b) Quantitative analysis of LFB- and Nissl-stained spared white matter area percentage in the R5mm, lesion core, and C5mm region over time. Spared white matter at the lesion core decreased at 1 day after SCI, dropped to the lowest point at 3 days after SCI, and remained stable from 7 to 14 days after SCI. ^*∗*^ Significantly different from the value before SCI. DC: dorsal column; LLWM: left lateral white matter; RLWM: right lateral white matter; LVWM: left ventral white matter; RVWM: right ventral white matter.

LFB and Nissl staining was used to test the post-SCI pathological processes and spared white matter at R5mm, the lesion core (LC), and C5mm (Figure 2(a)). LFB stained (light blue) myelin sheaths were evenly distributed throughout the white matter of the control rat spinal cord, and gray matter appeared in a typical H-shape and contained a large amount of purple neuronal Nissl bodies (Figure 2(a)). After SCI, the anatomic structure at the lesion core of the spinal cord was clearly changed, where a large amount of DCWM was lost, gray matter was severely transformed, and a large cyst formed at the spinal cord center (Figure 2(a)). This cyst was filled with a large amount of denatured and dead neurons and disintegrated axons and myelin debris, along with inflammatory tissue. Around the cyst, there remained spared white matter, especially at the ventral column. Additionally, different-sized cysts were observed in the spared white matter at R5mm, the lesion core (LC), and C5mm (Figure 2(a)). Similar to the morphological results, the MRI T2 image of the T7-T8 segment in control rats revealed a clear boundary between white matter and gray matter, gray matter in an H-shape, a medium-low signal for white matter, and a hyperintensity signal for surrounding cerebrospinal fluid (Figure 2(b)). At 1 day after SCI, a circular-like hypointensity signal was observed in the spinal cord close to the DC region in the T2 image at the lesion core (LC), indicating acute hemorrhage and a blurred boundary between white matter and gray matter. At 3 days after SCI, the hypointensity area at the lesion core (LC) remained. At 1 and 3 days after SCI, a circle-like hypointensity area was observed in the region close to the corticospinal tract at the dorsal column at R5mm and C5mm, indicating acute hemorrhage that spread along the rostral-caudal direction. At 7 days after SCI, hyperintensity signals appeared in the lesion core (LC), R5mm, and C5mm regions, indicating possible edema in these regions. At 14 days after SCI, the abnormal signal in the R5mm and C5mm regions was accompanied by a heterogeneous signal and a clearer boundary between white matter and gray matter. At 14 days after SCI, the hypointensity signal at R5mm and C5mm was accompanied by a heterogeneous signal, indicating a complication of hemorrhage, necrosis, and cyst denaturation, together with a blurred boundary between white matter and gray matter.

**Figure 3:**
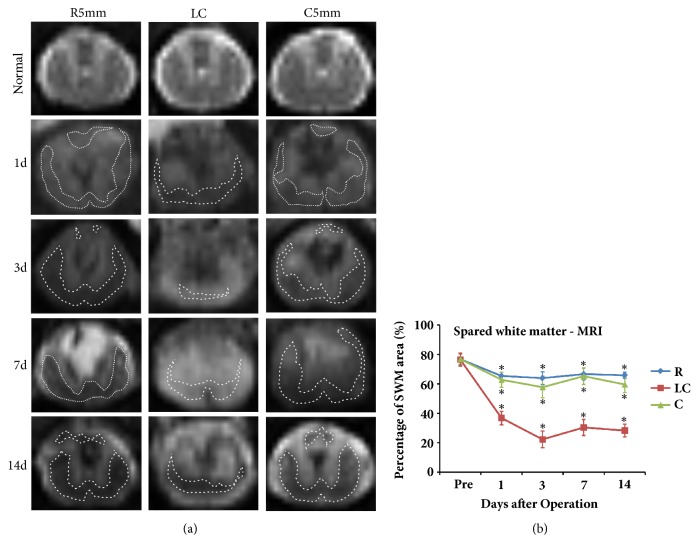
Structural image of MRI-T2 coronal sections at the lesion core (LC), R5mm, and C5mm segments at each time point after SCI. The variation of spared white matter area percentage over time was observed. (a) T2 images along the horizontal axis taken from the lesion core (LC), R5mm, and C5mm of each time point after SCI. The control rat has a clear boundary between white matter and gray matter at T7-8, gray matter is in an H-shape in the medium signal, white matter in the medium-low signal, and peripheral cerebrospinal fluid in a hyperintensity signal. At 1 day after SCI, a hypointensity signal was observed at the DC of the spinal cord, indicating acute hemorrhage and a blurred boundary between white matter and gray matter. At 3 days after SCI, the hypointensity signal at the lesion core remained, and a larger circle-like hypointensity signal shadow appeared in the region close to the corticospinal tract in the DC of R5mm and C5mm. At 7 days after SCI, a hyperintensity signal appeared in the lesion core (LC), R5mm and C5mm regions, indicating possible edema in these regions. At 14 days after SCI, the abnormal signal shadows shrank in the R5mm and C5mm regions, accompanied by a mixed signal and a clearer boundary between white matter and gray matter. The dotted line indicates spared white matter area. (b) Quantitative analysis of the variation in percentage of spared white matter area in the R5mm, C5mm, and lesion core (LC) regions over time, which is similar to the statistical findings for the morphological changes. ^*∗*^ Significantly different from the value before SCI: ^*∗*^*P *< 0.05.

**Figure 4:**
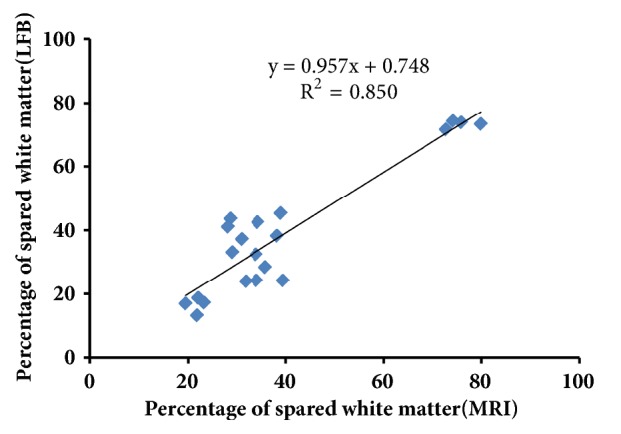
Correlation analysis of spared white matter (LFB) and spared white matter (MRI); the two sets of results were positively correlated.

The LFB-positive myelin sheath area percentage was calculated for different parts of the spinal cord (namely, R5mm, C5mm, and LC) at different time points after SCI. The results suggested that the LFB-positive area at the lesion core (LC) began to decrease at 1 day after SCI, reached the lowest point at 3 days after SCI, and became stable from days 7 to 14, but still being smaller than that before the operation (Figures 2(a) and 2(b)). The spared white matter area at R5mm and C5mm showed a similar variation trend over time. Compared with the white matter area before operation, the spared white matter area was significantly decreased at 1 day after SCI (Figure 2(c)); at 3, 7, and 14 days, the area continuously decreased, showing significant difference from the value before SCI (*P *< 0.05). Similar to the result by the quantitative analysis of LFB staining, the quantitative analysis of spared white matter area by MRI also revealed that, at 1 day after SCI, the spared white matter area at the lesion core (LC) began to decrease and reached the lowest point at 3 day (Figures 3(a) and 3(b)); at 7 and 14 days after SCI, this area became stable, but still smaller than before SCI (Figures 3(a) and 3(b)). The variation trend of the spared white matter area assessed by MRI at R5mm, the lesion core (LC), and C5mm over time was similar to that of LFB-positive myelin sheath area. According to the correlation analysis, the spared white matter area percentage measured by LFB was positively correlated with that measured by MRI (Figure 4, R^2^ = 0.8508), indicating that noninvasive MRI may work as a biomarker to determine spared white matter.

### 3.2. Spatiotemporal Variation of Myelinated Axons of White Matter Tract

**Figure 5:**
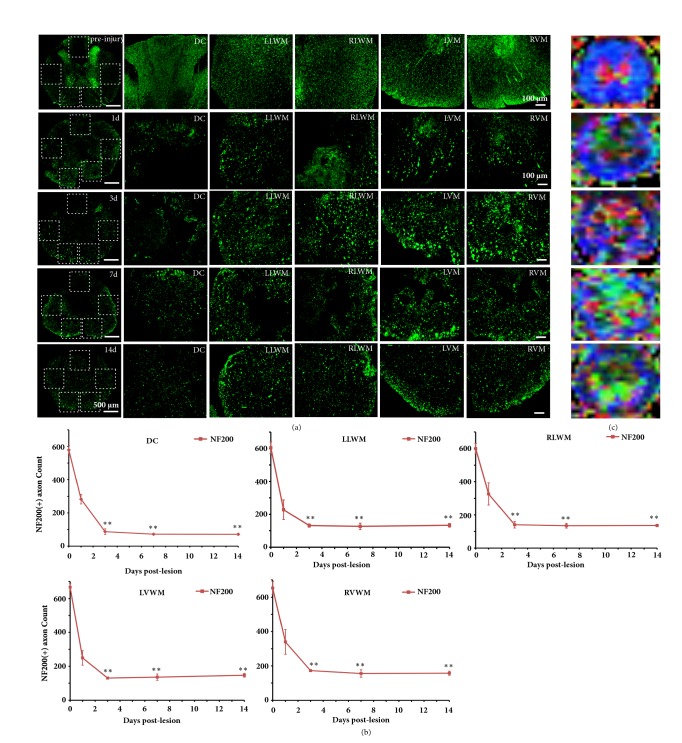
NF-200 immunofluorescent staining and the color FA maps at the lesion core segment showed the axonal variation in the SCI time course. (a) Before SCI and at 1, 3, and 14 days after SCI, axonal morphology of the DC, LLWM, RLWM, LVWM, and RVWM. The number of axons in the dorsal column (DC) region was lost quickly and severely at 1 and 3 days after SCI, and the axonal loss in the DC column was considerable, with spared axons characterized as disorganized, swollen, and ruptured. The axon loss and degeneration were continuously observed over the 14 days after SCI. (b) Before SCI and at different time points after SCI, axonal numbers in different regions. (c) Corresponding color FA maps before SCI and at different time points after SCI. ^*∗*^ Significantly different from those before SCI: ^*∗*^* P *< 0.05; ^*∗∗*^* P *< 0.01. Scale bar = 100 *μ*m.

To evaluate the pathological processes affecting axons in adult rat white matter tracts at different regions in the lesion core segment after SCI, coronal sections of the spinal cord at different time points after SCI were immunofluorescently stained for NF200. In the control rat spinal cord, NF200+ axons were regularly and evenly distributed throughout the white matter (Figure 5(a)). At 1 day after SCI, the number of NF200+ axons was greatly reduced in five white matter ROIs (regions of interest) in the spinal cord (Figures 5(a) and 5(b),* P *< 0.05, significant difference from before SCI), and spared axons were disordered, swollen, and collapsed. At 3 days after SCI, the number of NF200+ axons in the white matter tract continued to decrease, especially in the dorsal column, where the positive axon number was 86.33 ± 17.32 (Figures 5(a) and 5(b)), showing a significant difference from those numbers before SCI (*P *< 0.001). At 7 and 14 days after SCI, axon loss, swelling, and denaturation were still observed in the ventral column and lateral column (Figures 5(a) and 5(b)).

Figure 5(c) showed the corresponding color FA maps in lesion core level at different timepoints. According to the DTI data sets from the dorsal, lateral, and ventral regions of the spinal cord, we calculated the FA, MD, AD, and RD values in different ROIs (including the DC, LLWM, RLWM, LVW, and RVWM) at the lesion core (Table 1). The results showed that the DTI parameters of the uninjured group in various ROIs were basically consistent, and the FA, MD, and AD values in all ROIs sharply decreased at 1 day after SCI (*P* < 0.01). The FA and RD values reached nearly a minimum at 3 days after SCI and remained stable or slowly increased with time (except in the LVWM). On day 14 after SCI, the FA and AD values in each ROI were still significantly lower than those of the control group. However, the MD values in most ROIs returned to normal by 7 days after SCI. In contrast to other parameters, the RD values showed a significant increase rather than a decrease after SCI and remained stable with time. Remarkably, the RD in the DC area was observed to be significantly increased at 1 day after SCI (*P *< 0.001), while a marked increase in RD in the LWM and VWM was observed at 3 days after SCI. Similar to FA and AD, the most intense changes in RD values also occurred in the DC. Meanwhile, even at the last time point, the RD values in each ROI were significantly different from those in the control group (*P *< 0.05). The results also suggested that the longitudinal changes of FA and AD values in various ROIs were similar to the changes in the NF200+ axons.

### 3.3. Correlation between Spontaneous Recovery of Rat Hindlimb Motor Function and Spared White Matter after SCI

**Figure 6:**
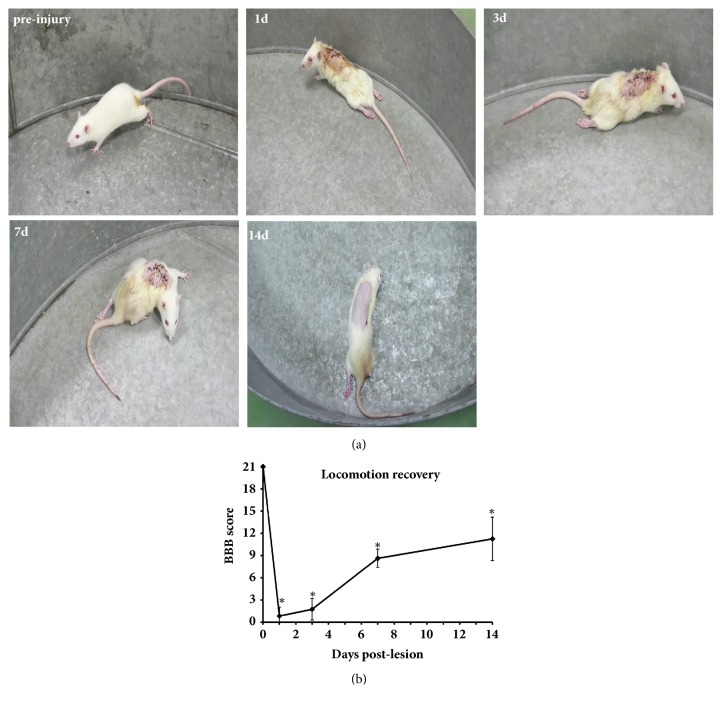
Open-field BBB scoring after severe spinal cord contusion. (a) Two observers blinded to the treatment carried out the BBB test on experimental rats in an open-field to evaluate the degree of recovery of locomotor function after SCI. The figure shows behavioral performance prior to the operation and at different time points after SCI. (b) BBB scoring at different time points after SCI. Hindlimb motor function was significantly reduced at 1 day after SCI and began to recover at 7 days after SCI. ^*∗*^ Significantly different from scores before SCI, ^*∗*^*P *< 0.05.

In the open-field test, the control rats displayed constant and coordinated gaits with constant toe clearance during walking, kept its paws parallel to the trunk when touching the ground, and stabilized its body and constantly stuck its tail up during movement (Figures 6(a) and 6(b)). At 1 day after SCI, the motor function of rat hindlimbs was largely lost, with bilateral BBB scores lowered to 1.75 ± 1.452 (*P *≤ 0.001, Figure 6(b)), and only slight movement of the hindlimb joints accompanied by partial flaccid paralysis** (**Figures 6(a) and 6(b)). Compared with the control rats, the bilateral BBB scores in rats at 3 days after SCI showed a statistical difference (*P *< 0.05), and the hindlimb motor function was barely improved** (**Figures 6(a) and 6(b)). At 7 and 14 days after SCI, the bilateral BBB scores were markedly increased, and the rat hindlimb motor function was recovered to some extent** (**Figures 6(a) and 6(b)), which was evidenced by continuous weight-supported plantar steps, constant coordinated movement of fore and hindlimbs, and joint rotation, or occasional dorsal stepping between touching the ground and lifting (Figure 6(b)). Collectively, motor function of rat hindlimbs was lost most severely at 1 and 3 days after SCI and partially recovered spontaneously at 7 and 14 days after SCI, but the bilateral BBB scores at 7 days and 14 days after SCI were still lower than those of the control rats (Figures 6(a) and 6(b);* P *< 0.05).

**Figure 7:**
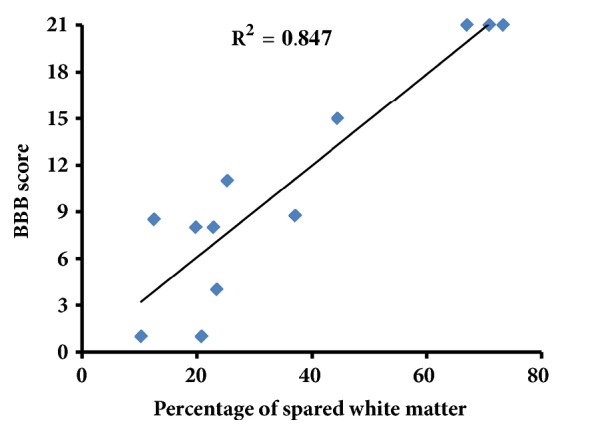
Correlation analysis of spared white matter at the lesion core (LC) and BBB scores of hind limb motor function. Except for the data at 1 day after SCI, the spared white matter area percentage at the LC is closely correlated with BBB scores (R^2^ = 0.846).

To explore the correlation between spared white matter percentage at the lesion core segment and the BBB scores of rat locomotion and hindlimb function in the open-field, a correlation analysis was conducted based on the experimental data at each time point. The linear correlation analysis showed that, except for the data at 1 day after SCI (trauma and plate removal had an impact on rat physical strength and physiological status), spared white matter at the lesion core segment and BBB scoring were highly correlated after SCI (R^2^ = 0.847, Figure 7).

## 4. Discussion

Using LFB myelin sheath staining and NF200 immunochemical staining, combined with in vivo MRI-DTI scanning and BBB behavior evaluation, the present study explored the variation in spared white matter after acute SCI and its correlation with behavioral function and spontaneous recovery. The aim was to reveal the variation trend of spared white matter in the early SCI stage, thus laying a theoretical foundation for selecting the intervention time window following acute SCI and establishing the potential repair protocol.

### 4.1. Spared White Matter

White matter loss is one of the major pathological processes after SCI and leads to functional disorder to different degrees [12]. The measurement of spared white matter area percentage by LFB and MRI showed similar statistical tendencies, both suggesting that the amount of the spared white matter at the lesion core segment decreased to the lowest point at 3 days after SCI, with the corresponding BBB score dropping to the lowest point (2.25 ± 0.38), except for the data at 1 day after SCI (trauma and plate removal had an impact on rat physical strength and physiological status), indicating that white matter loss caused the motor function loss of the hindlimbs in rat. The spared white matter area percentage, MRI quantitative analysis, and behavioral results in combination showed that, at 3 days after SCI, spared white matter decreased to the lowest value, accompanied by BBB scores at a relatively low level. Therefore, the first three days might be a key time window to protect the injured spinal cord in SCI therapy.

At 1 day after SCI, white matter fibers clearly decreased at R5mm and C5mm, indicating the spread of white matter damage and denaturation along the rostral-caudal axis, which is in agreement with Ward's results [12]. Later, the LFB-positive area percentage gradually increased, probably because of the spontaneous self-repair of myelin sheath and axonal sprouting. Remyelination is a normal responsive reaction of the myelin sheath to damage and can facilitate functional recovery to some degree [35].

### 4.2. Axon Damage

The white matter of the spinal cord contains many descending and ascending conduction bundle, which play an important role in motor and sensory functions. The axons of these conducting bundles are all NF200 positive. Therefore, we used immunofluorescence staining to observe the changes in the number of NF200 positive axons in spinal cord white matter at different time points of spinal cord injury to further understand the effects of spinal cord injury on different white matter regions. NF-200 immunofluorescent staining was used to observe the axonal variation in 5 ROIs at the lesion core segment after SCI. The number of axons in the dorsal column (DC) region was lost quickly and severely at 1 and 3 days after SCI, where most of the spared axons were fragmented due to the direct primary mechanical impact [23]. The amount of white matter in the ventral column (VC) region decreased at each time point after SCI caused by the counterblow, but to a smaller degree than in the DC, and the number of NF200+ axons in this region dropped to the lowest point at 3 days after SCI. This might be attributed to the secondary lesions, indicating that white matter damage degeneration spread along the dorsal-ventral axis [12].

Brennan et al. [16] observed the rodent spinal cord contused model using 16.4 T DTI and found that AD of the white matter was decreased at the lesion core segment, especially at the dorsal column region. In the present study, FA and AD in all ROIs were obviously decreased after SCI. The underlying mechanism may be attributed to multiple intracellular or extracellular factors. Extracellular factors may have included the initial primary mechanical damage that led to axonal tracts being stretched and transformed, which resulted in prolonged distances between axonal tracts, the breaking of axonal membranes, and myelin sheath loss and splitting that led to reduced diffusion of water molecules along the axial direction [23, 36]. Intracellular factors could have included cytoskeleton splitting, shortened distances between neural fibers, mitochondria edema, and increased Ca^2+^-activated proteinase that caused the degradation of neurofilament and tubulin [37, 38].

Previous studies indicated that the increase in RD values may be related to myelin disintegration [13, 39]. In this study, due to the initial mechanical damage that occurred in the DC of the spinal cord, the myelin sheath was disintegrated, while the RD values of the DC area also changed. It is worth noting that the RD values in LF and VF were significantly increased until 3 days after SCI, while the LFB staining results showed a large myelin loss in ventrolateral white matter at 3 days after SCI. This result suggested that the myelin disintegration of the ventrolateral white matter caused by the secondary injury was subsequent to the axon damage. Kim et al. [39] reported a similar result: serious axon damage was observed at the ventrolateral white matter in the epicenter of the spinal cord at 1 day after injury, while LFB staining showed essentially normal-appearing ventrolateral white matter myelin. Taken together, the DTI parameters show a high sensitivity to different pathological events. Meanwhile, the number of NF200+ axons and DTI results both suggested that the most severe lesion occurred in the dorsal column region, and some spared axons remained at the ventral and lateral columns. The spared axons might hold the key to motor function recovery.

### 4.3. Spontaneous Recovery of Hind Limb Motor Function

BBB behavior evaluation and quantitative analysis of spared white matter indicated that the motor function of the rats' hindlimbs after SCI spontaneously recovered to some extent; the percentage of the area of spared white matter at the lesion core segment was closely correlated with BBB scores (R^2^ = 0.846), which is consistent with previous results [11]. There are two potential reasons for the spontaneous functional recovery of the injured hindlimbs. On the one hand, as already reported, spared white matter plays a critical role in spontaneous recovery of rat hindlimb motor function and spared white matter is positively correlated with recovery degree [11, 13]. In the rat SCI model prepared by Loy et al. [40], the dorsal column was completely damaged, accompanied by a large reduction in the amount of white matter in the lateral column and ventral column; however, the open-field test showed that the rat hindlimb function was remarkably recovered. Some research has demonstrated that if the spared white matter is only 12-15% of the control rat, then the hindlimbs cannot move in a coordinated manner; however, if the spared white matter is 15-25% of the control levels, then forelimbs and hindlimbs can move in a coordinated manner [10, 11]. In the present study, we observed that at 14 days after SCI, the spared white matter at the lesion core segment was 27.43% ± 14.34%, corresponding to a BBB score of 11.25 ± 2.92, and the coordinated movement of the rats' fore and hindlimbs was observed. Moreover, the reticulospinal tract in the ventral and lateral white matter has been shown to be important for the recovery of rat motor function [10]. Our LFB staining results suggested that most of the spared white matter after SCI was located at the ventral column and the lateral column close to the anterior horn, where the reticulospinal tract resides. According to Schucht et al. [10], when the dorsal column white matter was injured and the ventral column white matter remained intact, movements related to touching the ground would not be influenced because the reticulospinal tract at the ventral lateral column or lateral column is important to the maintenance of these behaviors, while the corticospinal tract and rubrospinal tract at the dorsal column are essential for the more precise grid movements. Partial lesions of the spinal cord can be followed by spontaneous and often substantial functional improvements [41–43]. The mechanism underlying such recovery has been studied, and the occurrence of spontaneous sprouting of axons has been shown in a number of axonal tract systems, including the corticospinal tract [31, 44, 45]. These sprouting axons and spared spinal cord tissue form a neural circuit, leading to partial functional recovery [46]. On the other hand, the spontaneous recovery of hindlimb motor function may be attributed to the central pattern generator (CPG). The CPG neural circuit that initiates rhythmic movement is mainly located at T11-L2, while the CPG to coordinate left and right alternating activities is at T12-L4, whose neural fibers reside in the ventral white matter of the central canal [47, 48]. Previous research showed that when T13 was completely transected in cats, the hindlimbs could move alternately on a flat surface [49]. The neural fiber connection between the CPG and interneurons in the spinal cord can enable rhythmic movement even in the absence of descending movement control and ascending sensory input [47–49]. Therefore, the restoration of the rats' hindlimb functions in the present study may have been due to the effects of both spared white matter fibers and the CPG.

## 5. Conclusion

Using morphology, MRI-DTI, and behavior evaluation, the present study examined the spatiotemporal variation pattern of spared white matter after acute SCI and explored the correlation between spared white matter and motor function in rats. The results suggested that the first three days is a key time window for SCI diagnosis and treatment; spared white matter, especially in the ventral column, played a key role in rat motor function recovery. Additionally, DTI may be an important noninvasive technique to diagnose degree of acute SCI as well as a tool to evaluate functional prognosis.

## Figures and Tables

**Table 1:** Summary of the temporal changes at lesion core in FA, MD, AD, and RD for the various ROIs.

**ROI**	**DC**							
**DTI parameters**	**FA**		**MD**		**AD**		**RD**	
	mean	s.e.m	mean	s.e.m	mean	s.e.m	mean	s.e.m
**Pre-injured**	0.81	0.02	0.81	0.01	1.85	0.01	0.29	0.01
**SCI 1d**	**0.31** ^**∗∗∗**^	0.02	**0.64** ^**∗**^	0.03	**0.78** ^**∗∗∗**^	0.06	**0.56** ^**∗∗∗**^	0.02
**SCI 3d**	**0.28** ^**∗∗∗**^	0.01	**0.65** ^**∗**^	0.04	**0.76** ^**∗∗∗**^	0.06	**0.59** ^**∗∗∗**^	0.05
**SCI 7d**	**0.29** ^**∗∗∗**^	0.01	0.70	0.05	**0.94** ^**∗∗**^	0.09	**0.58** ^**∗∗∗**^	0.03
**SCI 14d**	**0.29** ^**∗∗∗**^	0.01	0.73	0.03	**1.02** ^**∗∗∗**^	0.07	**0.58** ^**∗∗∗**^	0.01

**ROI**	**LLWM**							
**DTI parameters**	**FA**		**MD**		**AD**		**RD**	
	mean	s.e.m	mean	s.e.m	mean	s.e.m	mean	s.e.m

**Pre-injured**	0.79	0.02	0.79	0.01	1.79	0.01	0.30	0.01
**SCI 1d**	**0.50** ^**∗∗∗**^	0.01	**0.62** ^**∗∗∗**^	0.02	**1.09** ^**∗∗∗**^	0.05	0.38	0.02
**SCI 3d**	**0.45** ^**∗∗∗**^	0.01	**0.67** ^**∗**^	0.03	**1.07** ^**∗∗**^	0.11	**0.46** ^**∗∗∗**^	0.02
**SCI 7d**	**0.46** ^**∗∗∗**^	0.01	0.71	0.03	**1.16** ^**∗**^	0.10	**0.48** ^**∗∗∗**^	0.02
**SCI 14d**	**0.47** ^**∗∗∗**^	0.01	**0.69** ^**∗**^	0.03	**1.15** ^**∗∗**^	0.08	**0.45** ^**∗∗∗**^	0.01

**ROI**	**RLWM**							
**DTI parameters**	**FA**		**MD**		**AD**		**RD**	
	mean	s.e.m	mean	s.e.m	mean	s.e.m	mean	s.e.m

**Pre-injured**	0.80	0.02	0.79	0.01	1.79	0.02	0.30	0.02
**SCI 1d**	**0.49** ^**∗∗∗**^	0.01	**0.62** ^**∗∗**^	0.02	**1.09** ^**∗∗∗**^	0.04	0.39	0.03
**SCI 3d**	**0.45** ^**∗∗∗**^	0.01	0.68	0.04	**1.08** ^**∗∗**^	0.08	**0.46** ^**∗∗**^	0.04
**SCI 7d**	**0.45** ^**∗∗∗**^	0.01	0.70	0.03	**1.13** ^**∗∗**^	0.09	**0.48** ^**∗∗∗**^	0.03
**SCI 14d**	**0.46** ^**∗∗∗**^	0.01	0.70	0.02	**1.18** ^**∗∗∗**^	0.05	**0.47** ^**∗∗**^	0.02

**ROI**	**LVWM**							
**DTI parameters**	**FA**		**MD**		**AD**		**RD**	
	mean	s.e.m	mean	s.e.m	mean	s.e.m	mean	s.e.m

**Pre-injured**	0.78	0.02	0.83	0.02	1.81	0.02	0.33	0.02
**SCI 1d**	**0.50** ^**∗∗∗**^	0.02	**0.62** ^**∗∗∗**^	0.02	**1.11** ^**∗∗∗**^	0.03	0.38	0.02
**SCI 3d**	**0.46** ^**∗∗∗**^	0.02	**0.69** ^**∗∗**^	0.01	**1.10** ^**∗∗∗**^	0.03	**0.49** ^**∗∗∗**^	0.03
**SCI 7d**	**0.47** ^**∗∗∗**^	0.02	**0.65** ^**∗∗∗**^	0.03	**1.01** ^**∗∗**^	0.10	**0.47** ^**∗∗**^	0.03
**SCI 14d**	**0.48** ^**∗∗∗**^	0.01	**0.64** ^**∗∗∗**^	0.02	**1.02** ^**∗∗∗**^	0.06	**0.45** ^**∗**^	0.01

**ROI**	**RVWM**							
**DTI parameters**	**FA**		**MD**		**AD**		**RD**	
	mean	s.e.m	mean	s.e.m	mean	s.e.m	mean	s.e.m

**Pre-injured**	0.78	0.02	0.82	0.02	1.82	0.02	0.31	0.02
**SCI 1d**	**0.51** ^**∗∗∗**^	0.02	**0.61** ^**∗∗∗**^	0.02	**1.09** ^**∗∗∗**^	0.04	0.37	0.03
**SCI 3d**	**0.47** ^**∗∗∗**^	0.02	**0.69** ^**∗**^	0.04	**1.10** ^**∗∗∗**^	0.12	**0.48** ^**∗∗∗**^	0.02
**SCI 7d**	**0.48** ^**∗∗∗**^	0.02	0.71	0.03	**1.14** ^**∗∗**^	0.10	**0.49** ^**∗∗∗**^	0.02
**SCI 14d**	**0.50** ^**∗∗∗**^	0.01	**0.67** ^**∗∗**^	0.02	**1.13** ^**∗∗∗**^	0.05	**0.44** ^**∗∗**^	0.01

^**∗**^ *P* < 0.05, ^**∗∗**^*P* < 0.01, and ^*∗∗∗*^*P* < 0.001.

## Data Availability

The data used to support the findings of this study are available from the corresponding author upon request.
